# Therapeutic dose prediction using score-based diffusion model for pretreatment patient-specific quality assurance

**DOI:** 10.3389/fonc.2024.1473050

**Published:** 2025-01-03

**Authors:** Xiuwen Yu, Jiabin Lin, Changfei Gong, Minhui Zhang, Xianyu Luo, Qiegen Liu, Yun Zhang

**Affiliations:** ^1^ Department of Electronic Information Engineering, Nanchang University, Nanchang, China; ^2^ Department of Radiation Oncology, Jiangxi Cancer Hospital & Institute, Jiangxi Clinical Research Center for Cancer, The Second Affiliated Hospital of Nanchang Medical College, Nanchang, China

**Keywords:** radiation therapy, score-based diffusion model, pretreatment patient-specific quality assurance, volumetric modulated arc therapy, dose prediction

## Abstract

**Objectives:**

Implementing pre-treatment patient-specific quality assurance (prePSQA) for cancer patients is a necessary but time-consuming task, imposing a significant workload on medical physicists. Currently, the prediction methods used for prePSQA fall under the category of supervised learning, limiting their generalization ability and resulting in poor performance on new data. In the context of this work, the limitation of traditional supervised models was broken by proposing a conditional generation method utilizing unsupervised diffusion model.

**Methods:**

A conditional generation method base on the score-based diffusion model was proposed, which employed diffusion model for the first time to predict the predict patients’ therapeutic doses (TherapDose). The proposed diffusion model TherapDose prediction method (DMTP) learns the data distribution of dose images. The data distribution contains the quantitative relationship between the radiotherapy dose (RTDose) derived from the VMAT plan files of the Treatment Planning System (TPS) and the measured Dose (MDose, i.e., TherapDose) obtained from the Dolphin Compass physical system. By sampling from the learnt distribution, efficient prediction of TherapDose was achieved. The training dataset comprises RTDose, and the MDose. The three-dimensional information of dose slice was utilized to predict TherapDose, aiming to enhance the accuracy and efficiency of TherapDose prediction. Root mean square error (RMSE), mean absolute error (MAE), and structural similarity (SSIM) metrics were leveraged to validate the effectiveness of the proposed method. Meanwhile, CT images were further added to test the impacts of CT images on the prediction effect of MDose.

**Results:**

The DMTP method has demonstrated superior performance in predicting TherapDose within key anatomical regions including the head and neck, chest, and abdomen, outperforming existing state-of-the-art methods by achieving high-quality predictions as measured across different evaluation metrics. It indicates that the proposed method is highly effective and accurate in its dose prediction capabilities.

**Conclusions:**

The proposed method has proven to be highly effective, consistently outperforming state-of-the-art techniques in MDose prediction across multiple anatomical regions and evaluation metrics. This method can serve as a clinical aid to assist medical physicists in diminishing the measurement workload associated with prePSQA.

## 1 Introduction

Contemporary radiation therapy stands as a paramount treatment approach for individuals diagnosed with cancer, encompassing intensity-modulated radiation therapy (IMRT) and volumetric-modulated arc therapy (VMAT) (1), which is required for over 50 percent of patients undergoing cancer treatment (2). However, quality assurance for radiotherapy planning is often a tedious, time-consuming, and complex task. Especially implementing pre-treatment patient-specific quality assurance (prePSQA) for individual patients. which arises a need for a more efficient, resource-friendly, and automated method for prePSQA in dose verification within radiotherapy centers (3, 4).

The advancement in machine learning (ML) and deep learning (DL), combined with their application in predicting QA outcomes, is expected to boost the effectiveness of patient–specific QA (5–10). Valdes et al. developed a Poisson regression model with Lasso regularization, successfully trained to forecast the gamma passing rate (GPR) of 3%/3 mm for a dataset consisting of 498 plans (5, 11). Subsequently, the validation of the predictive model was conducted across various institutions, employing diverse measurement approaches (6, 11). Granville employed support vector machines (SVMs) to categorize plans as cold, hot, or normal, utilizing parameters related to both plan complexity and accelerator performance (9, 10). Interian et al. developed a convolutional neural network (CNN) model (12), utilizing fluence maps from IMRT plans as its input, which exhibited comparable prediction accuracy to the previously established Poisson lasso model (8). A recently introduced prediction model based on ML utilizes a range of treatment plan parameters, including MLC apertures, gantry/collimator angles, couch positions and more, as input to predict dosimetric gamma passing rate (6, 13). This method eliminates the possibility of inaccuracies linked to the utilization of an unrealistic surrogate phantom or measurement instruments. Gong et al. achieved the successful development of a pretreatment prePSQA for VMAT, incorporating both DL and ML models based on dose–volume histograms (DVHs) (14). First, they applied a modified Res–UNet model to anticipate the distribution of the measured dose (MDose). Subsequently, they employed the XGBoost algorithm for the purpose of determining whether the result qualifies as a pass or not. Utilizing the MDose distribution enables the complete reconstruction of DVHs for all structures and facilitates the visualization of intricate 3D dose variations. Enabling more accurate detection of dose errors clinically relevant, outperforming the widely utilized gamma indexes (GIs) (14). In this paper, we refer to MDose–guided DVHs reconstruction for all structures as the patient therapeutic dose distribution (TherapDose) more appropriately.

With outstanding presentation capabilities, CNN–based architectures have achieved significant success in various medical applications. Owing to the intrinsic locality induced by convolution operations, these models frequently encounter challenges in explicitly modeling long–range dependency (15), and these models solely rely on low–dimensional dosimetry data, lacking the capability to capture the spatial information of volumetric doses. The radiation therapy administered to the head and neck (H&N) entails numerous micro–targets and organs at risk (OARs), the absence of local spatial information in the predictive networks could result in dosage errors. DVHs between the assessment of unapproved doses and the measurement of MDose have been incorporated into clinical practice. MDose can provide useful spatial information about complex dose distributions, based on which the DVH of all relevant structures can be completely reconstructed and detailed three–dimensional dose differences can be displayed. Therefore, it is more attractive for prePSQA to accept a full–volume dose image as input and then directly output a three–dimensional dose differential distribution.

Up to this point, the dose prediction methods applied to prePSQA fall within the realm of supervised learning. However, the generalization capability of supervised learning can be constrained when confronted with new domains or previously unseen data. Models may excessively fit the training data, leading to suboptimal performance on novel data. Unsupervised learning, on the other hand, proves valuable in unveiling latent patterns and structures within data, this aids in extracting essential information from the data, proving particularly beneficial for managing high–dimensional data and mitigating the intricacies of feature spaces. As the mainstream unsupervised models, a variety of deep generative models represented by the generative adversarial network (GAN) (16, 17), autoregressive model (18, 19), flow (20), variational autoencoder (VAE) (21, 22), denoising diffusion probabilistic model (DDPM) (23), and score–based generative model(SBGM) (24) have shown great advantages in generating high–quality samples. Within these models, utilizing a more efficient sampling approach, the score–based generative model further enhances the generative capabilities. There has been a recent surge in attention towards diffusion model and SBGM (23–25), with notable interest reflected in the works of Austin et al. (26–28). This increased attention has led to significant progress in advancing the modeling of continuous data. In the domain of speech synthesis, SBGM has demonstrated comparable human evaluation scores to state–of–the–art autoregressive models (29, 30). In the context of the class–conditional ImageNet generation challenge, SBGM has surpassed robust GAN baselines, as evidenced by superior FID scores (31, 32). In the realm of image super–resolution, SBGM has exhibited remarkable achievements in enhancing facial features, surpassing the performance of GANs (33).

The probability model of dose dataset is acquired by modeling the probability distribution of the dose dataset, and the TherapDose image is generated by sampling from probability distribution. Throughout the process of parameter fitting, the generative model has the capacity to acquire prior information. Inspired by this, simultaneously motivated by Gong et al.’s recent groundbreaking research in predicting 3D TherapDose (14), we proposed a novel unsupervised score–based diffusion model approach for predicting TherapDose by exploring its applicability within the realm of prePSQA. The model perturbs the data distribution through the introduction of Gaussian noise following the forward stochastic differential equation (SDE), leading to an known distribution. The relationship between noise disturbed data distributions with different noise levels, is learnt by a neural network. While training, deep priori information is acquired using images with multi–channel. During the prediction phase, the objective of incorporating the learned prior information as a constraint in the data consistency term of an optimization problem, which follows the least–squares method in iterative reconstruction, is to attain the optimal solution for dose prediction. Qualitative and quantitative experimental results demonstrate that the DMTP network outperforms several representative methods in TherapDose prediction, providing more accurate predictions. Specifically, the contributions of this work are summarized as follows:

- For the first time, the diffusion model “DMTP” is introduced in the field of radiotherapy for pre–treatment dose prediction, iteratively refining the target dose image through denoise, and generating samples from the data distribution.
- A novel conditional generation approach is proposed, utilizing multi–channel information to train high–dimensional priors as conditional guidance for target images, thereby acquiring more valuable prior knowledge through multi–channel dose learning.
- The effectiveness of DMTP has been demonstrated on dose map datasets for head and neck, chest, and abdomen. The results indicate that the DMTP approach surpasses classic supervised models such as U–Net and Res–UNet, further enhancing the accuracy of prePSQA.

## 2 Materials and methods

### 2.1 The DMTP method based on score–based diffusion model

**Figure 1:**
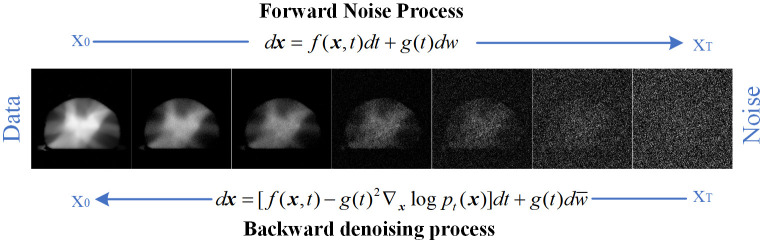
Forward and reverse processes of diffusion model.

For diffusion model, $p_{data}$ and $p_{T}$ denote the data distribution of interest (e.g., the distribution of dose image dataset) and the known distribution (e.g., Gaussian distribution). During the training process of diffusion model, the forward Stochastic Differential Equation (SDE) progressively injects Gaussian noise to transform complex data distributions into a known prior distribution (i.e., Gaussian noise), learning the characteristics of data distribution between two channels, which consist of RTDose and MDose. In the reverse SDE phases, the dual–channel input consist of RTDose and Gaussian noise map. The process of denoising Gaussian noise maps based on scores is essentially the process of sampling from the learned distribution. Ultimately the prediction process is achieved from the Gaussian noise map to the predicted dose (PDose, ideally the PDose is the MDose), illustrated as **Figure 1**. The DMTP method encompasses both the forward SDE and the reverse SDE.

#### 2.1.1 Forward SDE for model training

Forward SDE implements noise addition to dual–channel dose images. The continuous diffusion process (implemented by forward SDE), denoted as ${\left\{x\left(t\right)\right\}}_{t-0}^{T}$ with $x\left(t\right)\in {\mathbb{R}}^{n}$ (i.e., the two channel input of diffusion model in this work), Where t belongs to the interval [0, T], representing the temporal progression, and n signifies the dimension of the image. The forward SDE process can be formulated as follows:


(1) dx=f(x,t)dt+g(t)dw

Where $f:{\mathbb{R}}^{n}\mapsto {\mathbb{R}}^{n}$ represents the drift coefficient, while g:ℝ↦ℝ denotes the diffusion coefficient, and w represents a standard n–dimensional Brownian motion.

Different SDEs can be constructed by selecting various functions for both f and g . First, by choosing


(2) $$f=-\frac{1}{2}\beta \left(t\right)x,g=\sqrt{\beta \left(t\right)}$$

By using a monotonically increasing function of noise scale, denoted as 0<β(t)<1 , the Variance Preserving (VP)–SDE can be attained (34). In this situation, the signal magnitude gradually diminishes to 0, while the variances preserved to a fixed constant as t→∞ .

#### 2.1.2 Reverse–time SDE for dose prediction

By commencing with samples of $x\left(T\right)∼p_{T}$ and then reversing the SDE, samples of $x\left(0\right)∼p_{T}$ can be obtained, as described by Equation 3:


(3) $$dx=\left[f\left(x,t\right)-g{\left(t\right)}^{2}\nabla_{x}\log p_{t}\left(x\right)\right]dt+g\left(t\right)d\bar{w}$$

Where $\bar{w}$ represents a standard Wiener process as time progresses backward from T to 0, and dt corresponds to an infinitesimally small negative time step. After obtaining the $\nabla_{x}\log p_{t}\left(x\right)$ (i.e., score map) for each marginal distribution, for all time steps t . The reverse diffusion process can be derived using Equation 3 to sample from $p_{0}$ , achieving dose prediction.

#### 2.1.3 The score network for score estimation

**Figure 2:**
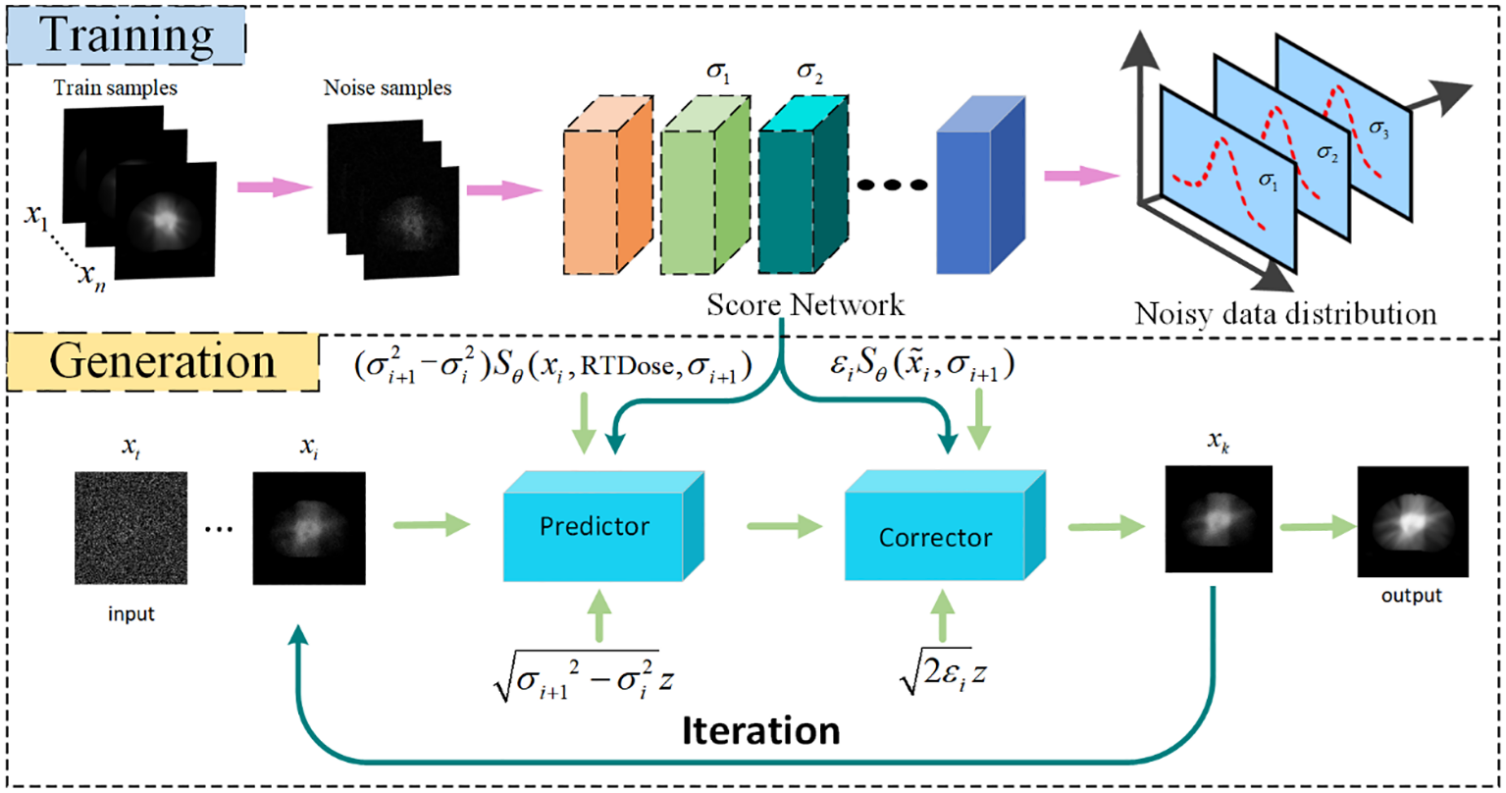
Flowchart of Dose prediction. Top: Training process for acquiring knowledge of the noise distribution through denoising score matching. Bottom: Reconstruction process, iteration using numerical SDE solver for achieving reconstruction. DC, data consistency.

**Figure 3:**
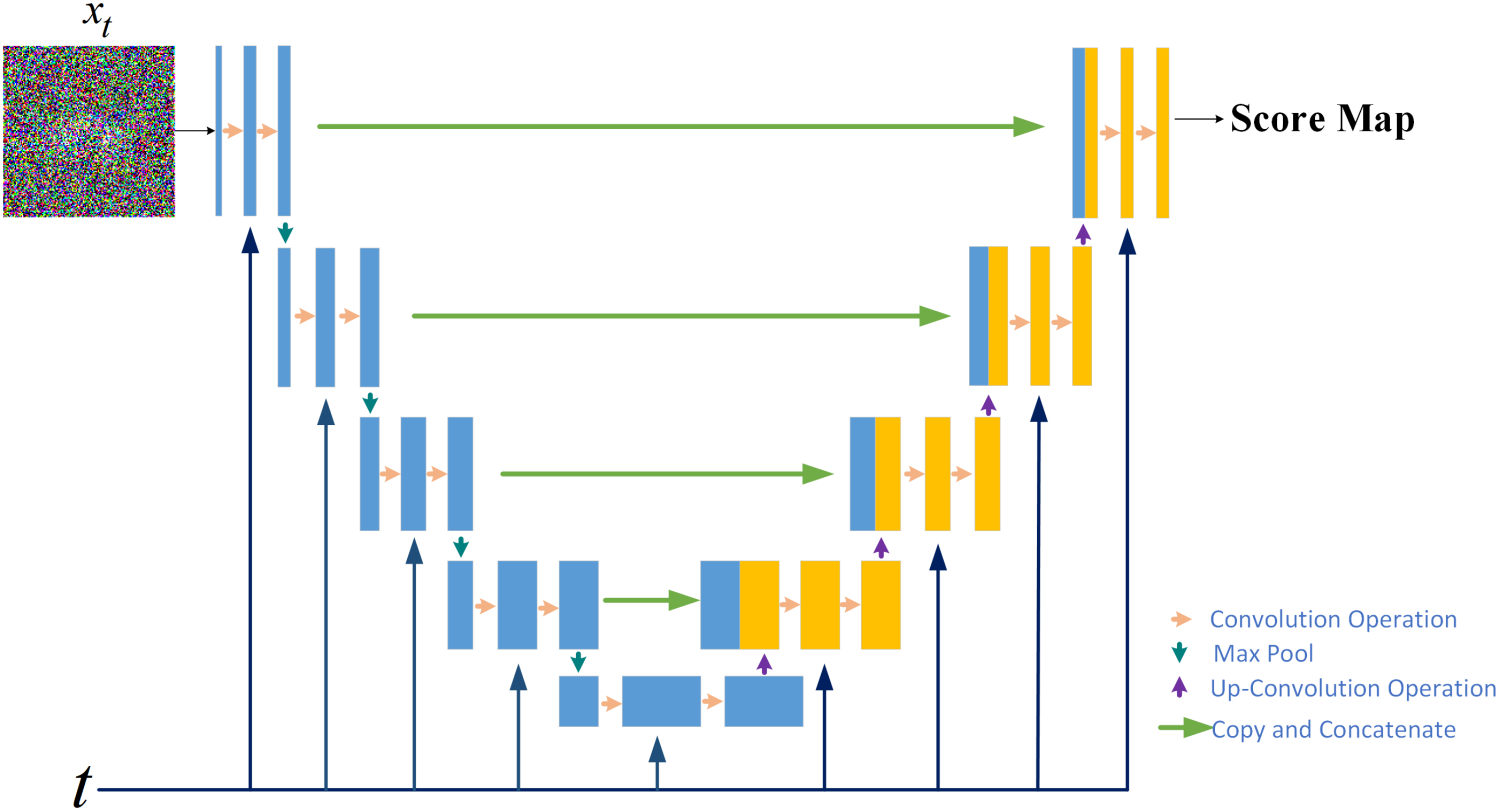
The score network structure for the score–based generative model.

The modelling of the probability distribution of the dose data can be achieved through score estimation (34). Score–based diffusion models approximate a distribution by training a time–dependent neural network $s_{\Theta }\left(x,t\right)$ to estimate the score of the distribution, shown in the upper part of **Figure 2**. Specifically, a U–Net serves as the neural network of the score–based generative model, shown in **Figure 3**. In the inverse SDE, the dose map disturbed by Gaussian noise and the RTDose function in a two–channel fashion as a network input, with feeding the temporal steps t incrementally into the network, as indicated by the deep blue arrows in **Figure 3**. By substituting the estimated scores into Equation 3, noise–disturbed dose images can be denoised (i.e., predicting MDose) given t by implementing score estimation via score networks.

### 2.4 The DMTP method specific description

During the training process, to address Equation 3, it’s essential to have knowledge of the score function $\nabla_{x}\log p_{t}\left(x\right)$ for all time steps. The unknown $\nabla_{x}\log p_{t}\left(x\right)$ in Equation 3 is substituted with $\nabla_{x}\log p_{t}\left(x_{t}|x_{0}\right)$ , which can be estimated by the score network. Where $\nabla_{x}\log p_{t}\left(x_{t}|x_{0}\right)$ signifies the gradient of the Gaussian perturbation kernel, with $x_{0}$ as the center. Throughout the training process, the parameters of the score network $S_{\Theta }\left(x_{t},t\right)$ are fine–tuned in accordance with Equation 6:


(6) $$\Theta *=\arg_{\Theta }\min{𝔼}_{t}\left\{\lambda \left(t\right){𝔼}_{x_{0}}{𝔼}_{x_{t}|x_{0}}[{\left\|S_{\Theta }\left(x_{t},t\right)-\nabla_{x_{t}\log}p_{t}\left(x_{t}|x_{0}\right)\right\|}_{2}^{2}\right\}$$

Once the score network is trained, MDose prediction can be accomplished by solving the reverse SDE. The reverse SDE presented in Equation 3 can be reformulated into Equation 7:


(7) $$dx=-d\left[\alpha^{2}\left(t\right)\right]s_{\Theta }\left(x_{t},t\right)+\sqrt{\frac{d\left[\alpha^{2}\left(t\right)\right]}{dt}d\bar{w}}$$

Based on the aforementioned knowledge, the iterative generation of PDose on the diffusion model comprises two main stages: prediction and correction, illustrated in the bottom section of **Figure 2**. The prediction of noise is derived by solving the reverse SDE numerically in the diffusion model. Subsequently, the direction of gradient ascent is adjusted through the application of the Langevin Markov Chain Monte Carlo algorithm (35),called correction. Within the prediction phase, Equation 8 is applied to forecast the data, and the target image, denoted as ${\hat{x}}_{i}$ , is produced based on the prior distribution that has been acquired through learning.


(8) $${\hat{x}}_{i}=x_{i+1}+\left(\alpha_{i+1}^{2}-\alpha_{i}^{2}\right)s_{\Theta }\left(x_{i+1},\alpha_{i+1}\right)+\sqrt{\alpha_{i+1}^{2}-\alpha_{i}^{2}z}$$

i=N−1,˙˙˙,0

Where ${\text{σ}}_{\text{i}}$ represents the noise scale, and i indicates the number of discretization steps for the reverse SDE, essentially denoting the number of iterations for dose prediction. The z follows a Gaussian white noise distribution with a mean of zero and a standard deviation of 1. During the correction step, we employ the correction algorithm as described in Equation 9 to rectify the direction of the gradient ascent.


(9) $${\tilde{\text{x}}}_{i}={\hat{\text{x}}}_{i}+\varepsilon_{i}S_{\Theta }\left({\hat{x}}_{i,}\alpha_{i+1}\right)+\sqrt{2\varepsilon_{i}z}$$

**Algorithm 1** presents the pseudo–code for the reconstruction algorithm, consisting of two loops. In the Training Process, the dual–channel data is input into the network to learn the dose data distribution between two channels. For the prediction process, the number of iterations (N) in the outer loop is determined by the discrete steps of the reverse SDE. M is the number of corrector steps. The inner loop is refined through annealing Langevin iteration.

Algorithm 1 **Algorithm 1:DMTP for iterative generation.**

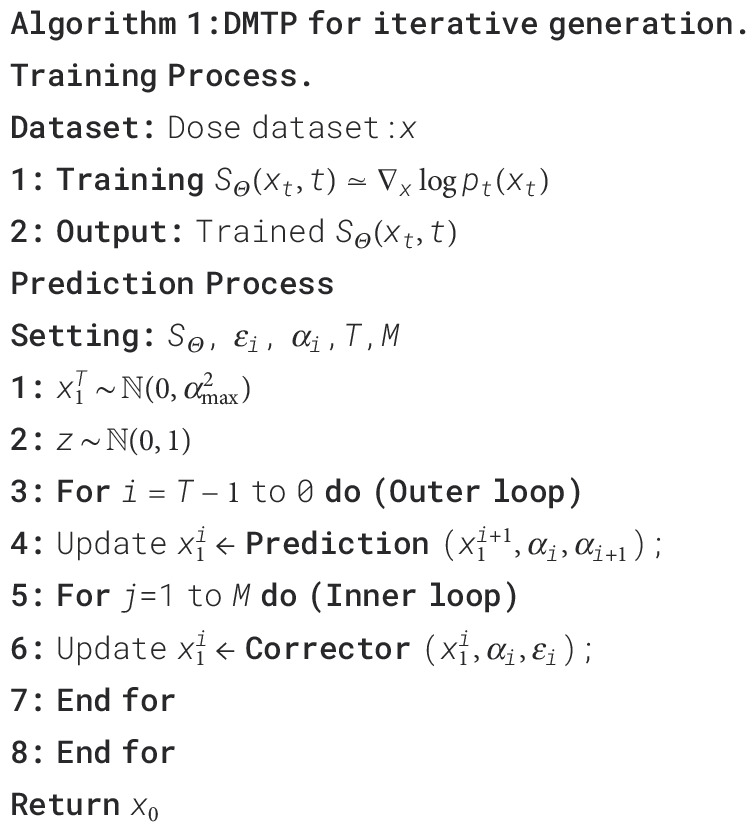

### 2.5 Data collection

This study has been approved by the medical ethics committee of our hospital (2022KY012) and conducted in accordance with the principles embodied in the Declaration of Helsinki, as well as local statutory requirements. The requirement for informed consent was waived by the ethics committee because of the study’s retrospective nature. And this study only uses imaging and plan data from the patient’s previous treatment for analysis and will not have any impact on the patient’s treatment. In addition, there is no commercial purpose or action in this study. Thereby informed consent from the patient is not required. Between 2018 and 2021, a cohort of 300 patients underwent VMAT. After integrating the data from head and neck (H&N), chest, and abdominal tumors to achieve a more comprehensive feature representation and enhance the predictive robustness during the training phase, a 4:1 ratio was subsequently applied to randomly select 240 cases for training purposes, while designating the remaining 60 cases specifically for testing. **Table 1** presents a summary of the clinical characteristics observed in these patients. CT scans were conducted with the Somatom Confidence imaging system (Siemens Healthcare, Forchheim, Germany). Senior radiation oncologists utilized magnetic resonance imaging and positron emission tomography images to aid in contouring the target volumes. The VMAT plans were created using the Monaco TPS (clinical version 5.11) employing the Monte Carlo algorithm. These plans utilized a 6–MV photon beam and were administered on an Elekta Infinity machine equipped with an agility MLC. Each plan underwent optimization to achieve coverage of the target volume that is deemed clinically acceptable, while also ensuring the sparing of OARs. The Dolphin–Compass system (version 3.0, IBA Dosimetry, Schwarzenbruck, Germany) was utilized for the measurement of prePSQA. Rigorous commissioning of the Dolphin–Compass system, which included validating accuracy for array measurement, beam modeling, and dose reconstruction, was carried out beforehand in accordance with the manufacturer’s specified standards.

**Table 1:** Clinical attributes of cancer patients included in this investigation.

Characteristics	Sample number	Percentage
Gender,no.		
Male	191	63.7%
Female	109	36.3%
Age (years)		
<20y	25	8.3%
20y–60y	96	32.0%
>60y	179	59.7%
Cancer sites		
H&N	115	38.3%
Chest	87	29.0%
Abdomen	98	32.7%

### 2.6 Experiment setup

To assess and measure the effectiveness of the current DMTP method, three alternative DL methodologies were employed for comparison, namely U–Net (3, 36, 37), Res–UNet (14), and TransQA (38). The approaches can be outlined as follows: (1) U–Net, a conventional encoder–decoder network, has been recently applied in dose prediction. (2) ResU–Net, utilized for prePSQA, predicts dose distribution using input data comprising CT structure, and RTDose obtained from TPS, along with dose distributions measured by the Dolphin Compass system and ArcCHECK–3DVHs system. (3) TransQA, this network combines a Transformer based on a self–attention mechanism with an improved U–Net for predicting TherapDose of prePSQA. In this study, we assessed the proposed model through two input modalities: A) predicting from RTDose to TherapDose (RTDose → TherapDose), B) predicting from both RTDose and CT to TherapDose (RTDose + CT → TherapDose).

In this study, we employed the Structural Similarity Index (SSIM) and Mean Absolute Error (MAE) as the evaluation metrics for assessing the accuracy of prePSQA. SSIM was chosen due to its ability to capture the structural similarity between the predicted and actual dose distributions, providing a comprehensive assessment of the spatial and structural fidelity. Meanwhile, MAE quantifies the absolute differences between predicted and actual dose values, offering precise insights into prediction accuracy. To ensure equity and uniformity, irrespective of the prediction method employed in this investigation, the data grouping, preprocessing, and training procedures remained consistent.

**Figure 4:**
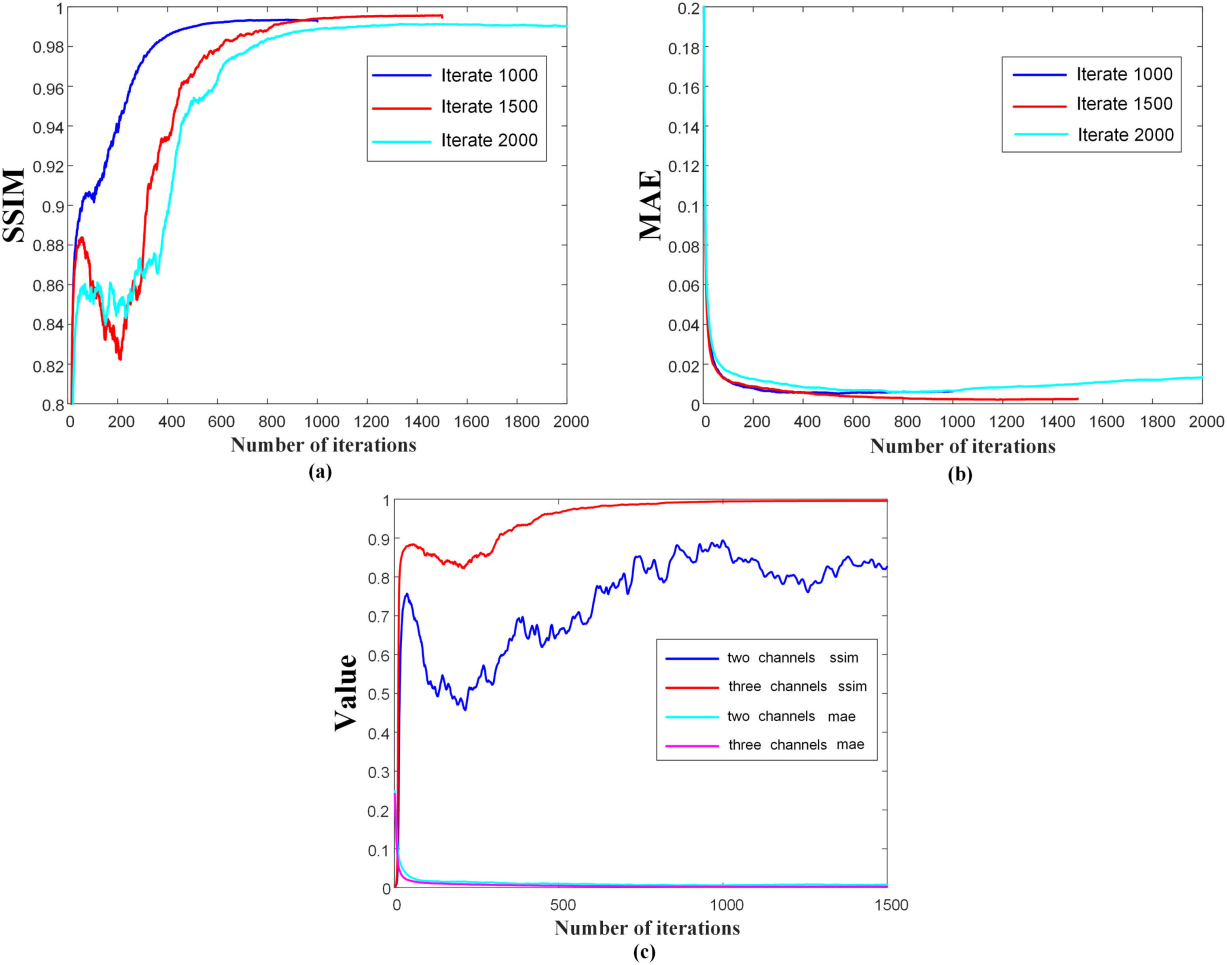
Iteration curves of the model with different iteration steps and different number of channels.

In this study, we performed four comprehensive ablation experiments to gain insight into the functioning of the proposed DMTP method and confirm its effectiveness under various parameter configurations. In order to analyze the influence of noise scale division on the structural characteristics of fractional network learning dose distribution, experiments were set up for different iterations (1000 steps, 1500 steps and 2000 steps) and the same dataset was used for training and testing. According to Part (b) of **Figure 4**, when the number of model iterations is 1000, the fractional network cannot fully learn the image distribution of the training dataset and reconstruct the dose image within a small noise scale. When the number of iterations is set to 2000, the extraction of image features during training is too high, and some unnecessary image structure details of the training set data are learnt by the model, which produces a pathological output during the test reconstruction, and the overfitting leads to the decline of the test index. Due to the fixed time of one iteration of the model, considering the cost of time computing power and the effect of image reconstruction, the model with a noise scale of 1500 is selected. Since the fractional network is a visual convolutional neural network, the pixels between different channels are summed and calculated with multiple convolutional kernels to obtain the output with different structural information, so controlling the data information input of each channel is of great significance for the model to learn the dose distribution. In order to determine the most suitable channel information input, the two–channel and three–channel mode control inputs were used for model training and testing. We trained in 2–channel mode, and in order to compare the impact of adding CT mode on model performance, we increased the number of channels and added CT to another channel for training. Then we tried the model performance of the three–channel model without CT mode, and found that the model training effect of the three–channel model was better. And the DMTP starts sampling the image in the target distribution from the known prior distribution, i.e., the pure noise map, and the selection of the initial noise distribution can also affect the quality of the generated image. We set up different initial noise test models to better understand the sensitivity of the models to noise. The fractional network is based on the visual neural network of convolutional kernels to estimate scores, and the study of the number of convolutional kernels is also of great significance for predicting the reconstruction dose map. For the first convolution operation of the model, when the number of convolution kernels is small, the model cannot extract enough structural features for training, and the model training cannot converge. When the number of convolution kernels is large, the model is prone to overfitting and learning unnecessary structural features. At the same time, the number of initial convolution kernels is proportional to the number of model parameters, and the number of convolution kernels is 64 and 128, respectively, to compare the performance impact of the model, considering the computing power limitation and the training and testing time. The results show that a better effect can be achieved with a convolution kernel of 128.

## 3 Results

The quantitative results for all test cases are presented in **Table 2**. It is evident that our method surpasses other three methods in terms of three–dimensional dose distribution in two input modes of the three quantitative indicators. Among these, the DMTP method of mode A performed the best, with SSIM, RMSE and MAE of 0.9965, 0.0046 and 0.0017, respectively, and the efficacy of this model significantly surpassed that of U–Net and marginally exceeded that of Res–UNet. Moreover, in mode B, TransQA exhibits better performance than both U–Net and Res–UNet, it still does not exceed the DMTP. For input mode A, the RMSE and MAE values of DMTP are 56.96% and 54.79% lower than those of Res–UNet, respectively, and for mode B, the RMSE and MAE values of DMTP are 20.72% and 13.56% lower than those of TransQA, respectively.

**Table 2:** Comparison of predictions with three advanced methods in terms of SSIM, RMSE, and MAE (MEAN+STD).

Tasks	A:RTDose → TherapDose			B:RTDose+CT → TherapDose		
Metrics	SSIM	RMSE(%)	MAE(%)	SSIM	RMSE(%)	MAE(%)
U–Net	0.9721 ± 0.0227	2.0448 ± 2.0028	0.8226 ± 0.8374	0.9793 ± 0.0270	1.5367 ± 1.6036	0.5524 ± 0.6167
TransQA	0.9837 ± 0.022	1.2231 ± 1.4969	0.3999 ± 0.4908	0.9907 ± 0.1712	0.9792 ± 1.243	0.343 ± 0.4516
Res–UNet	0.9846 ±0.017	1.0750 ± 1.2734	0.3896 ± 0.4593	0.9874 ± 0.0184	1.1144 ± 1.2607	0.4049 ± 0.4708
DMTP	0.9965 ± 0.0031	0.4626 ± 0.3292	0.1761 ± 0.1298	0.9949 ± 0.0039	0.7763 ± 0.6268	0.2965 ± 0.2453

**Figure 5:**
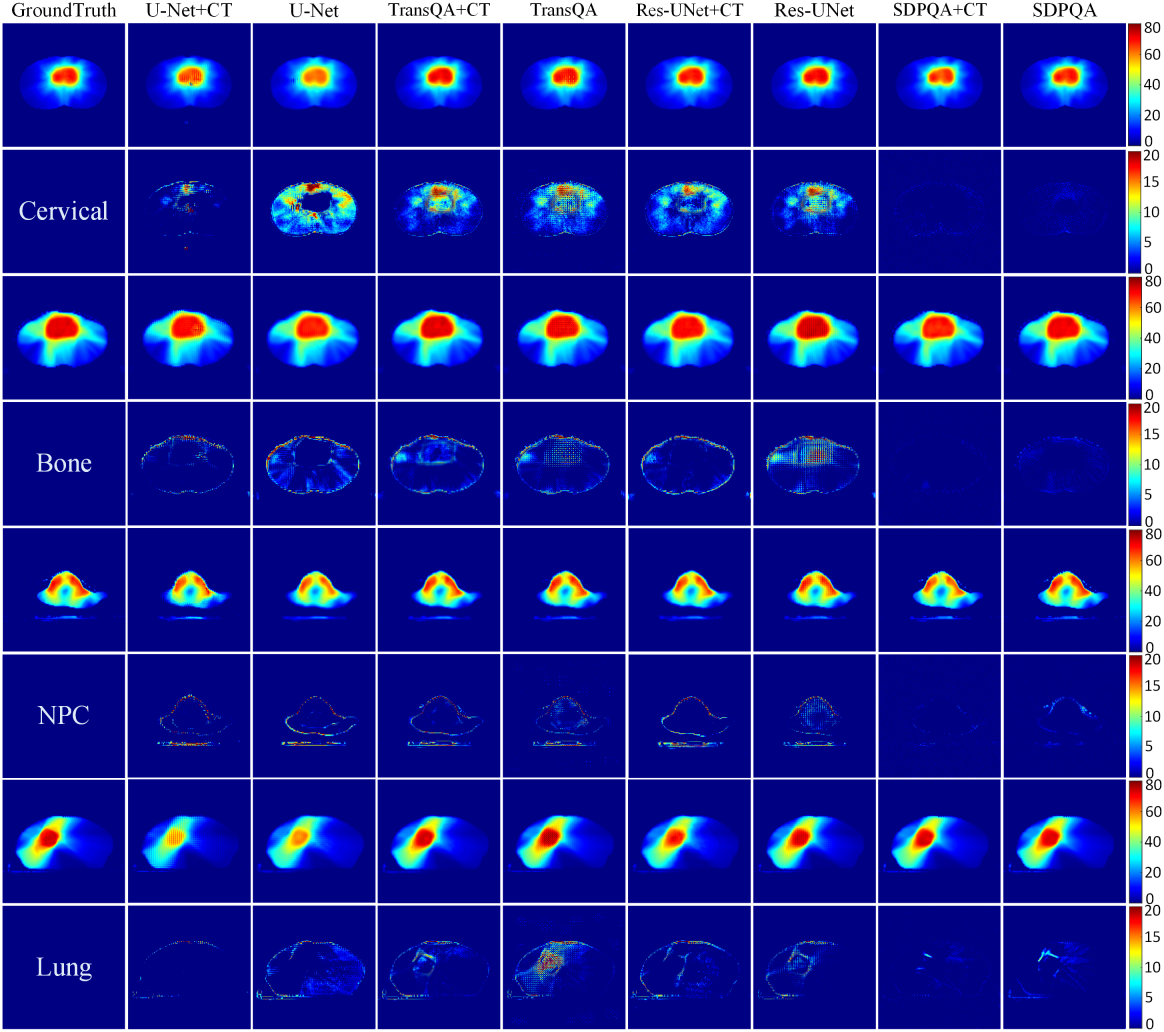
Visualization outcomes of various comparison methods. The first, third, fifth, and seventh rows illustrate the PDose distributions across four distinct patients, i.e., Cervical case, bone case, NPC case and lung case. The second, fourth, sixth, and eighth rows portray the disparities between the ground truth and the predictions.

**Figure 5** displays the qualitative outcomes of four distinct cancer scenarios: cervical cancer, bone metastasis, nasopharyngeal cancer, and lung cancer. The first, third, fifth, and seventh rows depict the transverse dose distribution, while the second, fourth, sixth, and last rows illustrate the disparities between the actual and predicted values. In visual observation, the dose difference plot of DMTP was significantly better than that of U–Net, TransQA and Res–UNet, and in addition, the results with CT input were similar to those without CT input, and even from the picture, the residual plot with CT input was better than without CT.

**Figure 6:**
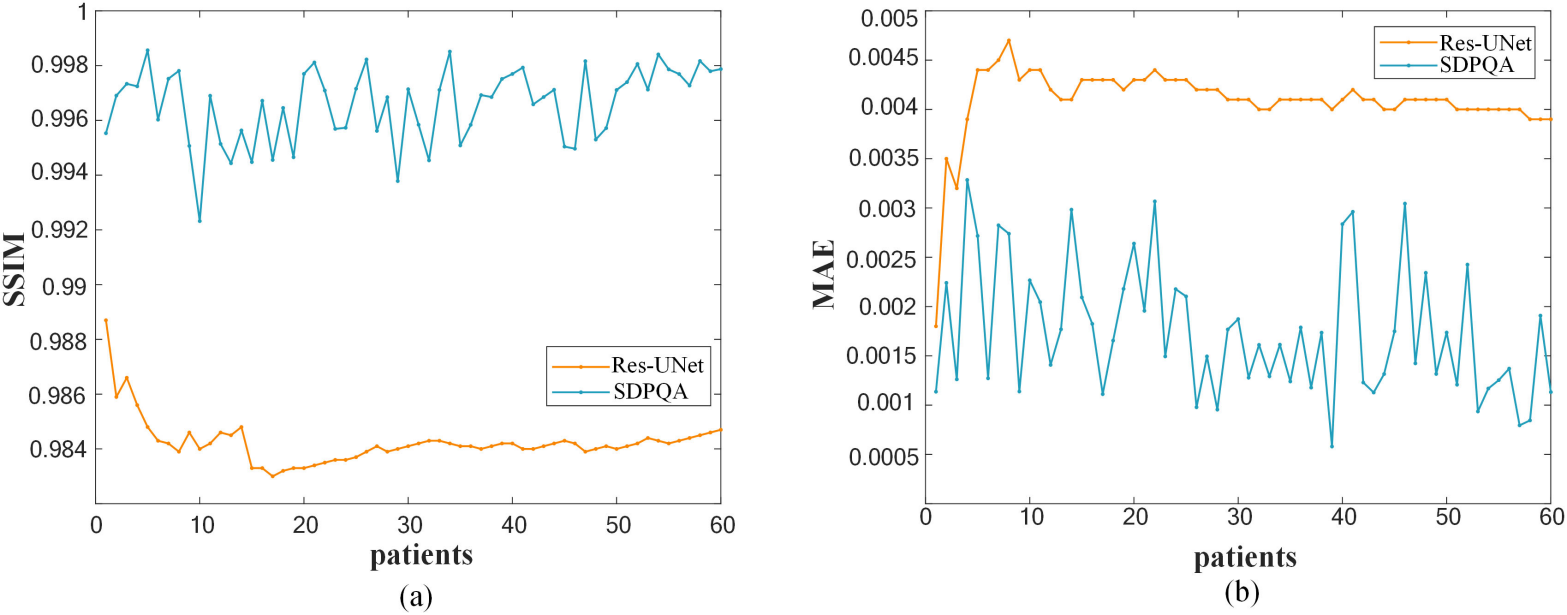
**(A)**Comparison of SSIM for each patient. **(B)**Comparison of MAE for each patient.

**Figure 7:**
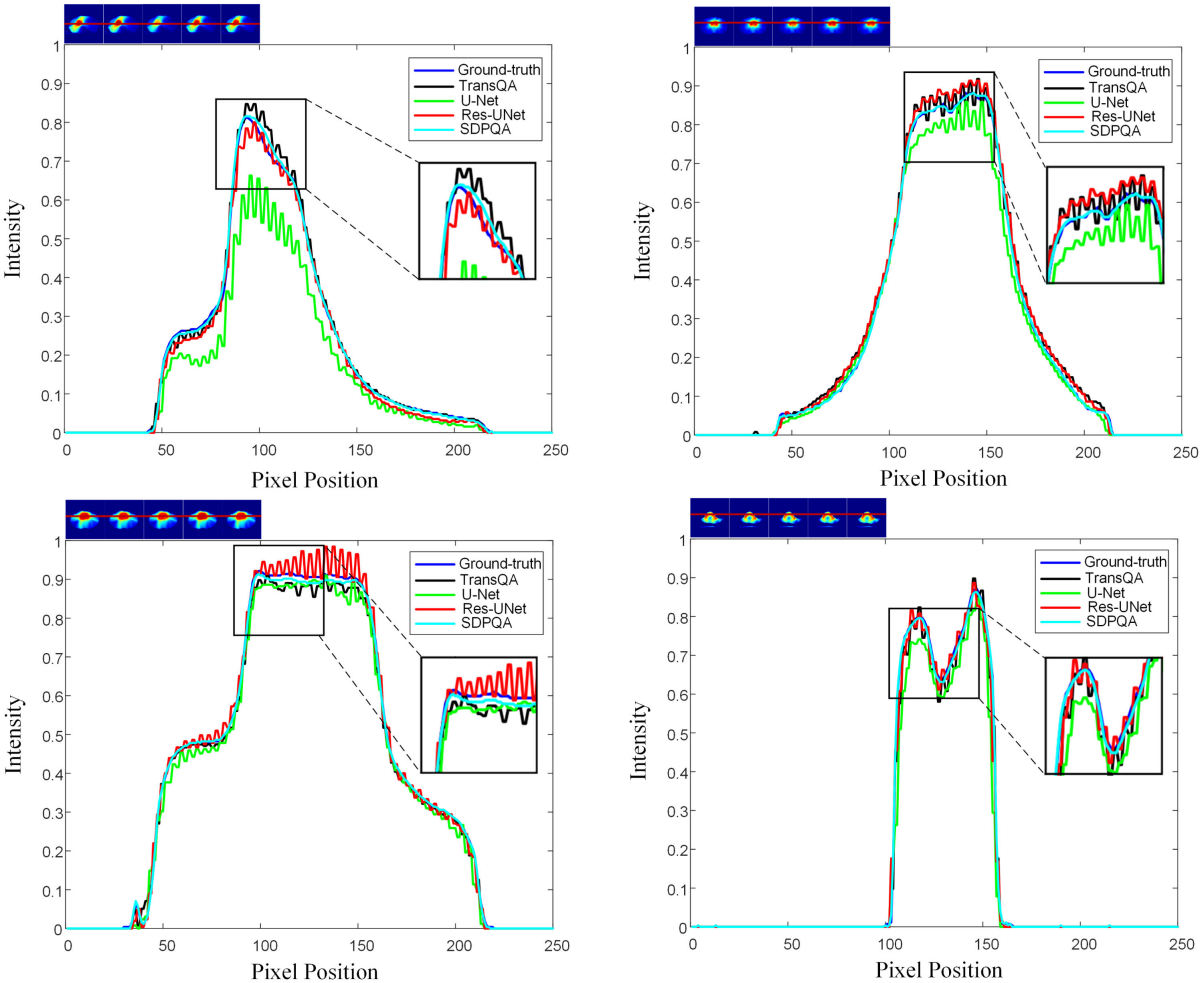
The horizontal dose difference profiles at various cancer sites for comparison between measured dose and predicted dose using four different methods. The “blue line” represents the measured dose., the “black line” represents the TransQA predicted prePSQA dose, the “green line” represents the U–Net predicted prePSQA dose, the “red line” represents the Res–UNet predicted prePSQA dose, and the “cyan line” represents the DMTP predicted prePSQA dose.

**Figure 6** illustrates a comparison of SSIM and MAE predicted by Res–UNet and DMTP in 60 test patients. We found that the current DMTP was superior to Res–UNet in SSIM and MAE in all patients. In addition, **Figure 7** displays the transverse profiles of four distinct cancer patients. The local amplification results show that the curve matching results of DMTP and MDose are very close, and the other methods are highly volatile. Compared with Res–UNet, the matching results of the DMTP method are closer. In other words, the current method offers more favorable benefits compared to others.

**Figure 4** shows the iteration curves of the model for different iteration steps and different channel numbers, and **Figures 4A, B** show that the model with 1500 iterations is better than 1000 and 2000 times. **Figure 4C** shows that the model performance tends to decrease with the increase of the number of iteration steps in the 2–channel mode, while the model training is more stable in the 3–channel mode. Therefore, we chose a three–channel mode with an iteration of 1500 steps.

**Table 3** shows the performance of DMTP at different noise scales and convolution kernel counts. The quantitative results show that the network performance is slightly improved when the noise scale is 6e*–1 in the non–CT input mode, while the SSIM is almost the same when the noise scale is 6e*5 in the CT input mode, however, the MAE of the model is slightly lower when the noise scale is 6e*5. At the same time, we observe that as the scale of noise is added, the time required for the network becomes progressively longer, and the memory used by the model becomes larger. When the number of convolution kernels is 64, the SSIM without CT input is slightly better than that of the model with 128 convolution kernels, but its MAE will become higher, while in the CT input mode, when the convolutional kernel is 64, the SSIM of the model decreases significantly, and the MAE decreases slightly. After comprehensive consideration, we chose a noise scale of 6e*–1 and a convolution kernel number of 128 for experiment.

**Table 3:** The effects of different noise scales and number of convolution kernels on DMTP.

Metrics	A:RTDose → TherapDose			B:RTDose + CT → TherapDose		
	SSIM	RMSE(%)	MAE(%)	SSIM	RMSE(%)	MAE(%)
6e*–1	0.9965 ± 0.0031	0.4626 ± 0.3292	0.1761 ± 0.1298	0.9949 ± 0.0039	0.7763 ± 0.6268	0.2965 ± 0.2453
6e*2	0.9955 ± 0.0066	0.4780 ± 0.4524	0.1866 ± 0.1646	0.9914 ±0.0330	0.9746 ± 3.1825	0.4028 ± 1.5727
6e*5	0.9952 ± 0.0124	0.4790 ± 0.3115	0.1910 ± 0.1274	0.9948 ± 0. 0059	0.4859 ± 0.3778	0.2250 ± 0.1910
64	0.9971 ± 0.004	1.0407 ± 0.9947	0.3584 ± 0.3830	0.9919 ± 0.009	0.6503 ± 0.4467	0.2748 ± 0.2175
128	0.9965 ± 0.0031	0.4626 ± 0.3292	0.1761 ± 0.1298	0.9949 ± 0.0039	0.7763 ± 0.6268	0.2965 ± 0.2453

**Figure 8:**
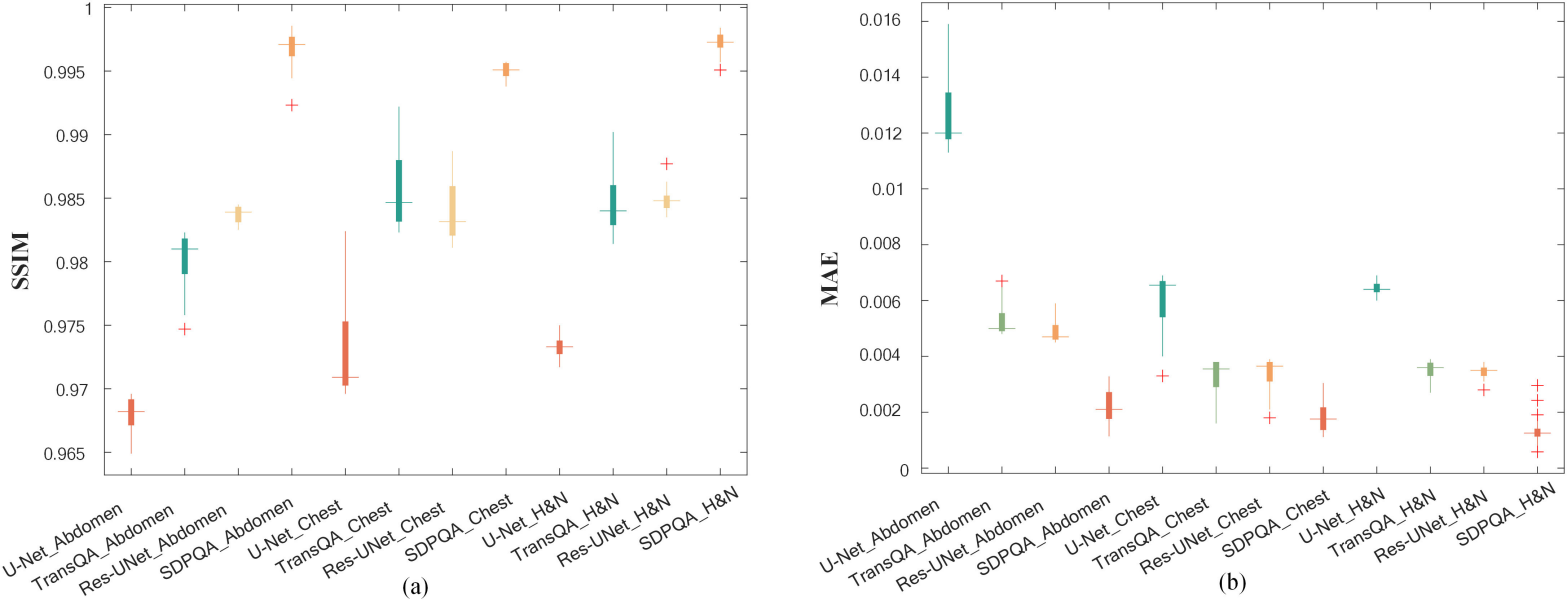
The SSIM and MAE values from three methods for abdominal cases, chest cases and H&N cases. The box represents the interquartile range (IQR), with the upper and lower boundaries of the box denoting the first quartile (Q1) and the third quartile (Q3), respectively, reflecting the interquartile range. The horizontal line in the middle of the box represents the median. The figure displays the distribution of experimental results for each group, as well as the detected outliers (marked with plus signs).

In addition, **Figure 8** shows box plots of SSIM and MAE values for abdominal cases, chest cases, and H&N cases for the four methods. It can be found that the other three methods have the worst effect in abdominal cases and the best effect in H&N cases, while DMTP has a more stable prediction effect on each case and has achieved very good results, and the numerical outcomes further show that the proposed DMTP surpasses other methods in predicting TherapDose distribution.

## 4 Discussion

In this study, we developed a new unsupervised dose prediction method based on a diffusion model, in which all three channel data are diffused into the noise during training to learn the gradient of the data distribution, and the score is matched by the score estimation, and the image is recovered from the noise by spreading backwards during the test phase. To the best of our knowledge, DMTP is the first unsupervised prePSQA prediction framework for TherapDose prediction based on diffusion models.

PrePSQA plays a crucial role as a validation stage within the IMRT/VMAT regimen. However, the tasks associated with prePSQA are intricate and demand a significant amount of time. In recent times, numerous researchers have introduced diverse approaches aimed at mitigating the complexity and time–consuming nature of prePSQA work (6–11, 13, 14, 36, 37). The unsupervised dose prediction network based on the diffusion model proposed in this study can predict the TherapDose distribution of multiple cancers based on TPS information. Gong et al. introduced a new method for VMAT prePSQA, incorporating a combination of DL and ML models based on the DVHs. In a similar fashion, they utilized TPS information to predict the dose distribution for prePSQA in cancer cases. Taking inspiration from the work of Gong et al., we enhanced the input data to enable predictions transitioning from RTDose to TherapDose (14). Jia et al. introduced an fGAN (39–42), which relied on radioluminescence imaging for the validation of radiotherapy dose. To evaluate the performance of our proposed method qualitatively and quantitatively, we compared DMTP with U–Net, TransQA, and Res–UNet. The discrepancy between the predictions of U–Net, TransQA, and Res–UNet in the high–dose area compared to MDose is evident from **Figure 5**. For cervical cancer patients, they have the worst prediction effect, while DMTP achieves very accurate prediction for all four cancer cases.

It is observed that the method in this paper has obvious advantages in predicting the main structural features after CT, but there is a large noise in the background, which may be the reason for the low overall index. This background noise is random in nature and is not correlated with the dose distribution. It interferes with the denoising score matching process of the diffusion model during training and inference, leading to increased noise in the final dose predictions. This contributes to the lower overall performance metrics, despite the improvement in capturing the main structural features provided by the inclusion of CT images. As a result, while CT images offer useful prior information, their random noise can adversely affect prediction accuracy in some instances.

Generating a predicted dose map from a pure noise map with a known distribution is a unique part of this work. According to **Table 3**, the choice of the initial noise scale for image generation can slightly affect the dose prediction, but the lower initial noise can speed up the iterative process. The results of CT showed that the generation stability of high noise was relatively improved after CT was added, suggesting that high initial noise could remove the instability factors in the image and improve the stability of pixel–level prediction of dose. It is speculated that high noise reduces the background noise introduced by CT and improves the stability of prediction. The number of convolutions at the beginning significantly affects the model’s ability to extract image features in the first step. The experimental data analysis in **Table 3** shows that a higher number of convolution kernels can yield more stable outputs, and empirically, the training of the model can be accelerated.

As for hyper–parameter configuration, we utilized a 3x3 convolutional kernel, a standard choice in convolutional neural networks (CNNs) In this work, known for its balance between computational efficiency and performance. This configuration was adopted in line with the default settings of Song et al. (24)’s diffusion model code. Given its widespread use and proven effectiveness in various diffusion models, we opted not to conduct an ablation study on kernel size. However, we acknowledge that exploring different kernel sizes may further optimize model performance, and this will be a focus of future investigations.

As can be seen from **Figure 6**, our DMTP method is better than Res–UNet for each case in the test set. As can be seen from **Figure 7**, the prediction results of U–Net, TransQA and Res–UNet are jagged and volatile, and there is a certain error with the prediction results of TherapDose, while the prediction results of our DMTP method almost match the prediction results of MDose, and there is no jagged plot line. This suggests that several other methods may have lost some details during training. As can be seen from **Figure 8**, the three methods such as U–Net are not as effective in abdominal cases, while DMTP has achieved very good predictive results in abdominal cases, H&N cases, and chest cases, even if the difference in divorce values is good.

Regarding iteration steps, they refer to the progressive refinement of the model from a pure noise distribution to the target data distribution. According to Song et al. (24), increasing the number of noise levels enhances the model’s capacity to learn a more accurate representation of the data space by improving denoising score matching. While a higher number of iterations can lead to improved performance, this also introduces a trade–off in terms of longer training times and increased computational cost during inference. Our study conducted ablation experiments to identify a balance between the number of iterations and computational efficiency as shown in **Figure 6**. Future work will delve deeper into optimizing iteration steps to minimize overfitting while maintaining computational feasibility.

Regarding evaluation metrics, although the DICE score is widely used to assess volumetric overlap, particularly in studies focusing on the Planning Target Volume (PTV) and Organs at Risk (OAR), we found SSIM and MAE to be more appropriate for our analysis. This is because our model focuses on accurately predicting the spatial details and structural features of dose distributions, which are better captured by these metrics. Furthermore, by reconstructing dose–volume histograms (DVHs) based on predicted dose distributions, as demonstrated in (14), we can more effectively visualize and quantify clinically significant dose variations, surpassing the capabilities of the commonly used gamma index (GI).

Additionally, as isodose lines are essential for clinical dose assessments, particularly in evaluating PTV coverage and ensuring proper dose delivery to the target while minimizing exposure to healthy tissues. However, this study primarily focuses on the overall accuracy of predicted dose distributions compared to actual distributions, using metrics like SSIM and MAE. Since our model assesses broader dose prediction accuracy rather than specific prescription doses around the PTV, isodose line analysis was not a key focus and falls outside the scope of this evaluation.

Unlike the end–to–end network, which uses a single network to encode and decode all features and make predictions, the diffusion model uses a t–dependent fractional network to learn the dose structure features of different sizes at different noise scales, which has higher stability. There is limited literature on directly predicting TherapDose from MDose. Nevertheless, extensive research has been conducted on dose reconstitution for automated planning purposes. Building upon Gong et al.’s work, we focused solely on utilizing RTDose from the TPS as input to train the DMTP network. The quantitative findings presented in **Table 2** also indicate that more precise predictions can be achieved using RTDose as the sole input data. In addition, our ablation experiments show that the number of iteration steps when generating the prediction results will also have an impact on the generation effect of the model, and the effect of model generation cannot be optimal when the number of iteration steps is small, and the model cannot converge well when the number of iteration steps is too large, thus affecting the prediction effect. We also compare the model effect of the two–channel and three–channel models, as shown in **Figure 4C**, in the two–channel mode, the model is unstable, and with the increase of the number of iteration steps, the model first rises and then decreases, while the three–channel mode will be more stable and finally converge. According to the results in **Table 3**, the model has better results when it starts to reconstruct the image with a small initial noise.

Owing to the inherent constraints associated with patient data and DL networks, certain discrepancies between predicted and measured results are unavoidable. Addressing these discrepancies in the future involves augmenting the dataset size or refining DL networks through optimization. Regarding the potential computational demands of multi–channel inputs and iterative reconstruction processes, we have implemented several optimization strategies within the model architecture to enhance computational efficiency and reduce inference time. For example, we adjusted the number of convolution kernels and the number of iterations to balance computational load and prediction accuracy. Additionally, during hyperparameter tuning, we weighed the trade–off between computational capacity and model precision to find the optimal balance. In future work, we plan to explore further optimization strategies, such as model compression techniques (e.g., pruning and quantization) and more efficient inference acceleration methods (e.g., GPU–based parallelization), to achieve faster real–time predictions. This will be a key focus of our future efforts to ensure the practical feasibility of the model in clinical settings. However, with the rapid advancement of computing and specialized hardware, improving the computational efficiency of the current DMTP method may become a less pressing issue. Nonetheless, the 3D TherapDose prediction of prePSQA, coupled with diffusion models, holds the potential to drive enhancements in prePSQA surpassing current or historical clinical practices. Furthermore, with ongoing refinement of our method, its adaptability to various radiotherapy scenarios is invaluable and plays a substantial role in clinical applications.

## 5 Conclusion

This research introduces a novel dose prediction method utilizing an unsupervised diffusion model. The experimental findings indicate a high concordance between the predicted TherapDose distribution and the actual scenario. DMTP emerges as a valuable tool, demonstrating effectiveness in dose validation and contributing to enhanced efficiency in prePSQA.

## Data Availability

The raw data supporting the conclusions of this article will be made available by the authors, without undue reservation.
